# Downregulation of P300/CBP-Associated Factor Protects from Vascular Aging via Nrf2 Signal Pathway Activation

**DOI:** 10.3390/ijms232012574

**Published:** 2022-10-20

**Authors:** Liqiang Qiu, Xiaoxiong Liu, Hao Xia, Changwu Xu

**Affiliations:** 1 Department of Cardiology, Renmin Hospital of Wuhan University, Wuhan 430060, China; 2 Cardiovascular Research Institute, Wuhan University, Wuhan 430060, China; 3 Hubei Key Laboratory of Cardiology, Wuhan 430060, China

**Keywords:** P300/CBP-associated factor, vascular aging, senescence, nuclear factor erythroid-2-related factor 2, oxidative stress

## Abstract

Increasing evidence has shown that vascular aging has a key role in the pathogenesis of vascular diseases. P300/CBP-associated factor (PCAF) is involved in many vascular pathological processes, but the role of PCAF in vascular aging is unknown. This study aims to explore the role and underlying mechanism of PCAF in vascular aging. The results demonstrated that the expression of PCAF was associated with age and aging, and remarkably increased expression of PCAF was present in human atherosclerotic coronary artery. Downregulation of PCAF could reduce angiotensin II (AngII)-induced senescence of rat aortic endothelial cells (ECs) in vitro. In addition, inhibition of PCAF with garcinol alleviated AngII-induced vascular senescence phenotype in mice. Downregulation of PCAF could alleviate AngII-induced oxidative stress injury in ECs and vascular tissue. Moreover, PCAF and nuclear factor erythroid-2-related factor 2 (Nrf2) could interact directly, and downregulation of PCAF alleviated vascular aging by promoting the activation of Nrf2 and enhancing the expression of its downstream anti-aging factors. The silencing of Nrf2 with small interfering RNA attenuated the protective effect of PCAF downregulation from vascular aging. These findings indicate that downregulation of PCAF alleviates oxidative stress by activating the Nrf2 signaling pathway and ultimately inhibits vascular aging. Thus, PCAF may be a promising target for aging-related cardiovascular disease.

## 1. Introduction

Age-related pathological changes of the vasculature play an essential role in the morbidity and mortality of the elderly [1]. In epidemiological studies, vascular aging is the main risk and driving factor of age-related cardiovascular disease [2]. Therefore, to develop novel therapeutic interventions to prevent age-related vascular disease, it is important to understand the cellular and molecular mechanisms of vascular aging.

Vascular aging is a special type of body aging, which could affect the occurrence, progression and severity of various cardiovascular diseases [3]. The occurrence of vascular senescence is closely related to the cell structure changes and dysfunction caused by the senescence of endothelial cells (ECs) located in the intima of the vessel wall. Studies have shown that senescent vascular endothelial cells are mostly accompanied by the accumulation of reactive oxygen species (ROS) and increased oxidative stress [4,5]. Excessive and continuous oxidative stress increased endothelial permeability, promoted leukocyte adhesion and changed endothelial gene expression, thereby causing endothelial dysfunction and being conducive to the development of vascular aging [6,7]. Senescent vascular cells have the general characteristics of aging, such as enhanced β-galactosidase activity; increased expression of aging-related proteins p53, p21 and p16; and decreased cell proliferation activity [8]. In addition, senescent vascular cells have active secretory functions, namely, senescence-associated secretory phenotype (SASP), which is mainly characterized by increased production of inflammatory cytokines and chemokines [9]. SASP factors could induce senescence and functional damage of adjacent vascular cells, further promoting the progress of vascular senescence [10,11]. Therefore, effective inhibition of vascular cell senescence is of great significance to the prevention and treatment of vascular-senescence-related diseases.

Nuclear factor erythroid-2-related factor 2 (Nrf2) is an evolutionarily conserved redox-sensitive transcription factor, which coordinates the antioxidant response by regulating the transcription of antioxidant factors, including heme oxygenase-1 (HO-1), NAD(P)H:quinone oxidoreductase 1 (NQO1), superoxide dismutase (SOD) and catalase (CAT) genes, thereby affecting the process of aging [12,13]. In young vasculature, this sensitive response of Nrf2 could alleviate oxidative stress and reduce the damage of vascular cells and macromolecules caused by elevated ROS levels [14]. The induction of Nrf2 also showed effective anti-inflammatory [15] and pro-angiogenesis effects [16]. There is strong evidence that Nrf2 activity decreases with vasculature aging, which will contribute to the further progress of vascular senescence [3,17].

Previous reports demonstrated that histone acetyltransferase (HAT) activity and histone acetylation level are closely related to longevity [18]. P300/CBP-associated factor (PCAF), also known as lysine acetyltransferase 2B, is a transcriptional adaptor protein and HAT [19]. As a member of the GCN5-related N-terminal acetylation transfer enzyme family, PCAF is mainly involved in the transcriptional regulation of genes through acetylation of histones and non-histones, and plays an important role in cell proliferation, differentiation, apoptosis, tumorigenesis and invasion [20,21]. Recent studies have shown that PCAF is a key regulator of arteriogenesis [22]. In addition, PCAF plays a pivotal role in vascular intimal neoplasia by regulating vascular inflammation [23]. We also found that PCAF has a significant role in regulating the inflammatory and function of vascular smooth muscle cells [24]. However, the potential role of PCAF in vascular aging has not been reported. Therefore, we tried to clarify the specific role and potential mechanism of PCAF in vascular aging.

## 2. Results

### 2.1. PCAF Is Highly Expressed in the Coronary Arteries of Older Patients as Well as in Coronary Arteries with Atherosclerosis

**Figure 1:**
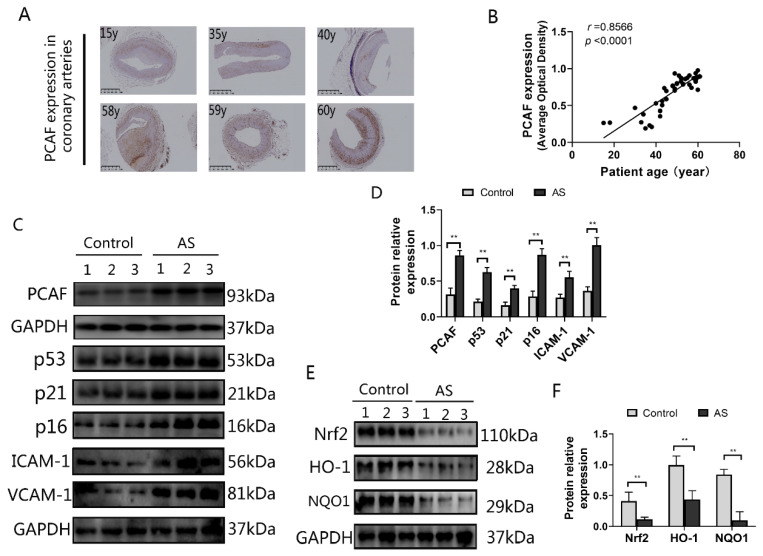
PCAF is highly expressed in coronary arteries of elderly patients and human atherosclerotic vessels. (**A**) All specimens were obtained from the proximal right coronary artery of the patient, and the expression of PCAF in human coronary arteries was detected by immunochemistry. The representative slides are shown; the scale bar is 1 mm, 2×. (**B**) The relationship between patient age and PCAF expression in human coronary arteries (*n* = 44). (**C**,**D**) The coronary intima produced “atherosclerotic plaque” as the criterion for judging atherosclerosis (AS). Western blot detects PCAF expression and aging-related protein (p53, p21, p16, ICAM-1 and VCAM-1) levels in human coronary arteries (*n* = 3). (**E**,**F**) Nrf2 signal pathway related protein level was detected by western blot (*n* = 3). ** *p* < 0.01 vs. the control group.

To explore the relationship between vascular aging and PCAF, we separately detected the expression of PCAF and aging-related markers in the coronary arteries. As shown in Figure 1A,B, the PCAF expression level was positively correlated with the age in these patients. Atherosclerosis is a disease related to vascular aging, and it is easier to detect vascular cells with an aging phenotype at the site of atherosclerosis [25,26,27]. Therefore, we tested PCAF and aging-related proteins expression in human coronary atherosclerotic plaques. Compared with healthy coronary arteries, the expression of PCAF was remarkably increased in atherosclerotic samples (Figure 1C,D). At the same time, senescence-related markers p53, p21, p16, ICAM-1 and VCAM-1 also increased significantly in atherosclerotic samples (Figure 1C,D). Furthermore, the expression of Nrf2, HO-1 and NQO1 were all downregulated in atherosclerotic samples (Figure 1E,F). All these results indicated that the upregulation of PCAF was closely related to vascular aging.

### 2.2. Downregulation of PCAF Inhibits AngII-Induced Senescence of ECs

**Figure 2:**
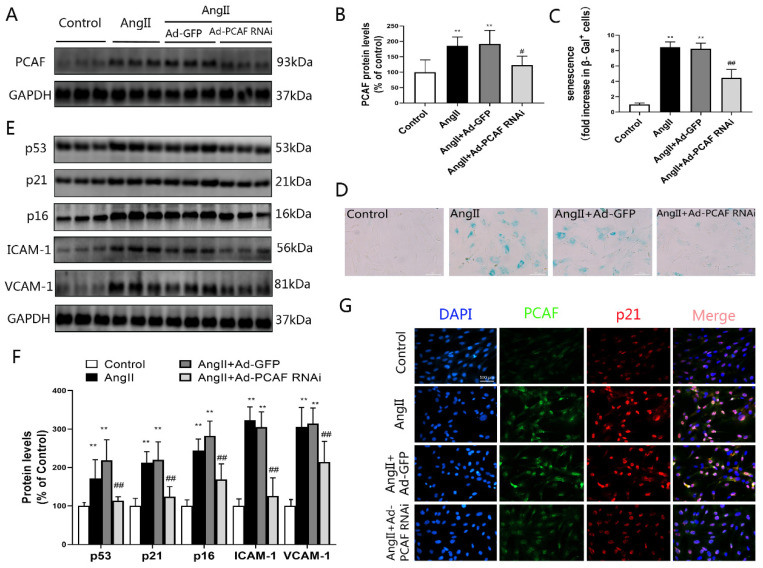
Downregulation of PCAF inhibits AngII-induced senescence of ECs. (**A**,**B**) Western blot to determine the expression of PCAF in each group (*n* = 6). (**C**,**D**) β-galactosidase staining to detect senescence of ECs; Senescent VSMCs were marked in blue, the scale bar is 100 μm, 400× (*n* = 5). (**E**,**F**) p53, p21, p16, ICAM-1 and VCAM-1 protein expression (*n* = 6). (**G**) Detection of PCAF (green) and p21 (red) levels by immunofluorescence double staining; the scale bar is 100 μm, 400× (*n* = 3). ** *p* < 0.01 vs. the control group; # *p* < 0.05, ## *p* < 0.01 vs. the AngII induction with Ad-GFP group (AngII + Ad-GFP group).

As shown in Figure 2A,B, the expression of PCAF in AngII–pretreated ECs was significantly increased, while Ad-PCAF RNAi transfection could effectively downregulate PCAF expression in ECs. SA-β-gal staining results showed that β-galactosidase activity in ECs was significantly enhanced after AngII induction, while downregulation of PCAF could inhibit this enhancement (Figure 2C,D). Interestingly, downregulation of PCAF also effectively inhibited the enhancement of AngII-induced p53, p21, p16, ICAM-1 and VCAM-1 expression (Figure 2E,F). Immunofluorescence double staining also showed that the co-expressions of PCAF and p21 upregulation were induced by AngII and disappeared when PCAF was silenced (Figure 2G). These results indicate that downregulation of PCAF could partially antagonize AngII-induced senescence in ECs.

### 2.3. The PCAF Inhibitor Garcinol Significantly Attenuates the AngII-Induced Vascular Aging Phenotype in Mice

**Figure 3:**
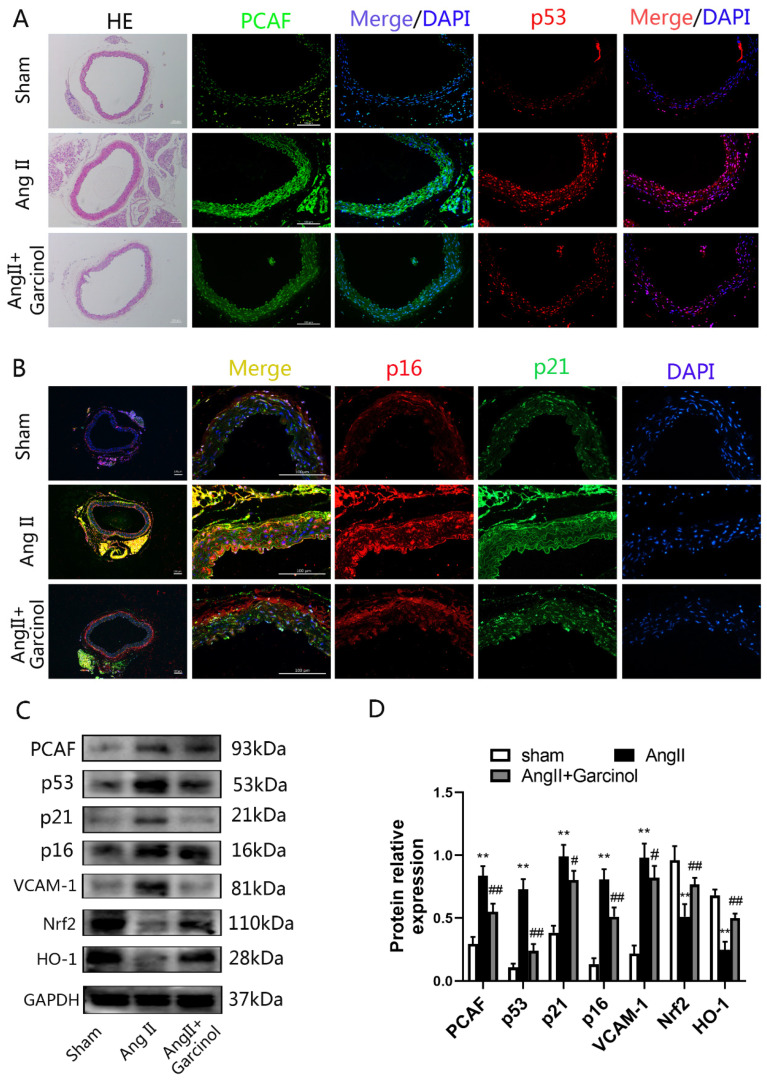
The PCAF inhibitor garcinol significantly attenuates the AngII-induced vascular aging phenotype in mice. (**A**) H&E staining to observe the vascular structure—the scale bar is 100 μm, 100× (*n* = 3); immunofluorescence staining to detect the expression of PCAF (green) and p53 (red)—the scale bar is 100 μm, 200× (*n* = 3). (**B**) Detection of p16 (red) and p21 (green) expression in mice thoracic aorta by double immunofluorescence staining; the scale bar is 100 μm, 400× (*n* = 3). (**C**,**D**) Western blot detects the expression of PCAF, aging-related proteins (p53, p21, p16, VCAM-1 and ICAM-1) and Nrf2 signaling pathway proteins in mice thoracic aorta (*n* = 6). ** *p* < 0.01 vs. the Sham group; # *p* < 0.05, ## *p* < 0.01 vs. AngII group.

Garcinol, a PCAF inhibitor, is widely used to inhibit PCAF in vivo [28,29]. Continuous pumping of Ang II into the abdominal cavity was used to construct vascular aging model in mice, and garcinol was injected into the abdominal cavity for treatment. After AngII injection, the expression of PCAF in the thoracic aorta of mice was increased and the expression of vascular aging-related proteins (p53, p21, p16 and VCAM-1) was also improved. However, treatment of garcinol could significantly ameliorate these aging phenotypes (Figure 3A–D). In addition, the expression of Nrf2 was remarkably inhibited in the AngII model group and HO-1 expression was significantly decreased compared with that in the control group, while garcinol treatment could restore the expression levels of Nrf2 and HO-1 (Figure 3C,D).

### 2.4. Downregulation of PCAF Attenuates Oxidative Stress in AngII-Induced ECs and Vascular Tissue in Mice

**Figure 4:**
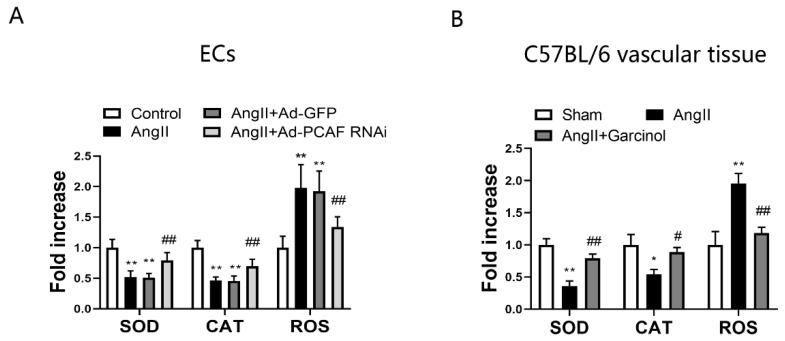
Downregulation of PCAF attenuates the levels of oxidative stress related markers in AngII-induced ECs and vascular tissue in mice. (**A**) Commercial kits to detect SOD, CAT and ROS activity in ECs (*n* = 5). ** *p* < 0.01 vs. the control group; ## *p* < 0.01 vs. AngII + Ad-GFP group. (**B**) Commercial kits to detect SOD, CAT and ROS activity in vascular tissue of mice (*n* = 5). * *p* < 0.05, ** *p* < 0.01 vs. the sham group; # *p* < 0.05, ## *p* < 0.01 vs. AngII group.

It is well known that oxidative stress plays an important role in promoting the process of vascular aging [5,30]. To clarify the effect of downregulation of PCAF on oxidative stress in vascular cells and vascular tissue, we detected the levels of SOD, CAT and ROS in ECs and mouse thoracic aortic vascular tissue, respectively. The results showed that the levels of SOD and CAT were markedly decreased after AngII induction in ECs, while downregulation of PCAF significantly increased the levels of SOD and CAT compared with those in the AngII + Ad-GFP group (Figure 4A). In addition, downregulation of PCAF remarkably decreased the level of ROS. Similar results were present in the mice thoracic aorta vascular tissue (Figure 4B). It is suggested that downregulation of PCAF could significantly alleviate AngII-induced oxidative stress injury in vivo and in vitro.

### 2.5. Nrf2/HO-1 Mediates the Anti-Senescence Effect of PCAF Downregulation

**Figure 5:**
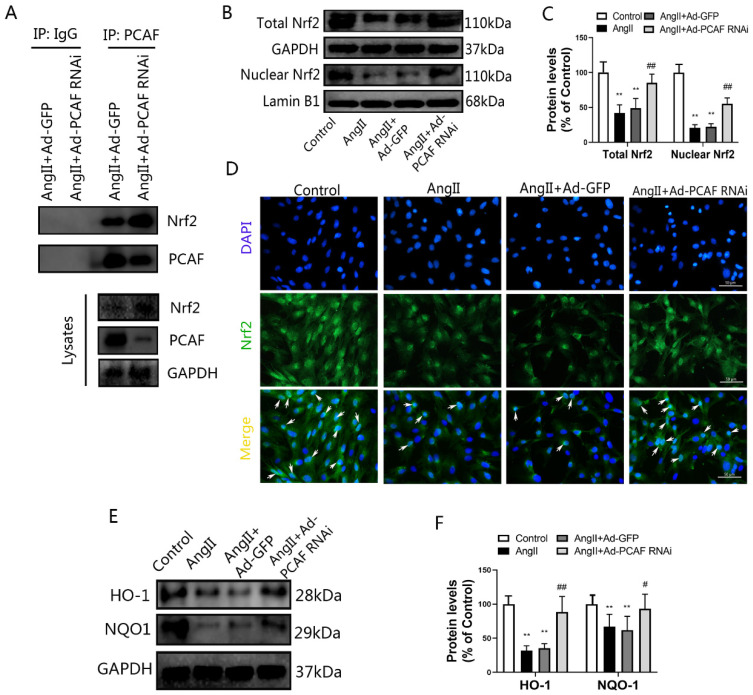
Nrf2/HO-1 mediates the anti-senescence effect of PCAF downregulation. (**A**) ECs cells were lysed for immunoprecipitation (IP) assays, and PCAF or Nrf2 were detected by Western blot in the precipitates pulled down by antibody against PCAF or normal IgG. Input indicates the cellular lysate before adding antibodies (*n* = 3). (**B**,**C**) The total expression of Nrf2 and the expression of Nrf2 in nucleus, detected by western blot (*n* = 6). (**D**) Immunofluorescence to detect the distribution of Nrf2 (green); arrows represent nuclear translocation of Nrf2; the scale bar is 50 μm, 400× (*n* = 3). (**E**,**F**) HO-1 and NQO-1 protein expression (*n* = 6). ** *p* < 0.01 vs. the control group; # *p* < 0.05, ## *p* < 0.01 vs. AngII + Ad-GFP group.

The antioxidant activity mediated by the Nrf2/HO-1 pathway plays a critical role in aging [31]. Upon activation, Nrf2 was transferred to the nucleus and could promote the transcription of antioxidant factors, thereby exerting anti-senescence effects. To clarify the connection between PCAF and Nrf2, cell lysates were immunoprecipitated with anti-PCAF antibodies. Co-immunoprecipitation results indicated that there was an interaction between PCAF and Nrf2, and this interaction was stronger after PCAF was downregulated (Figure 5A). Next, we detected the expression and distribution of Nrf2 in ECs. The results showed that compared with the control group, the total Nrf2 expression and nuclear Nrf2 distribution were significantly reduced after AngII treatment, while downregulation of PCAF reversed these changes (Figure 5B,C). Immunofluorescence staining further confirmed this result (Figure 5D). Further, we investigated the expression of Nrf2 downstream antioxidant factors, and the results demonstrate that the expression of HO-1 and NQO1 were significantly reduced after AngII induction, while downregulation of PCAF could inhibit the reduction induced by AngII (Figure 5E,F). These findings indicate that PCAF could interact with Nrf2, and downregulation of PCAF could preserve the activation of Nrf2 and subsequent expression of its target genes.

### 2.6. Nrf2 Silencing Aborted the Anti-Senescence Effect of Downregulation of PCAF

**Figure 6:**
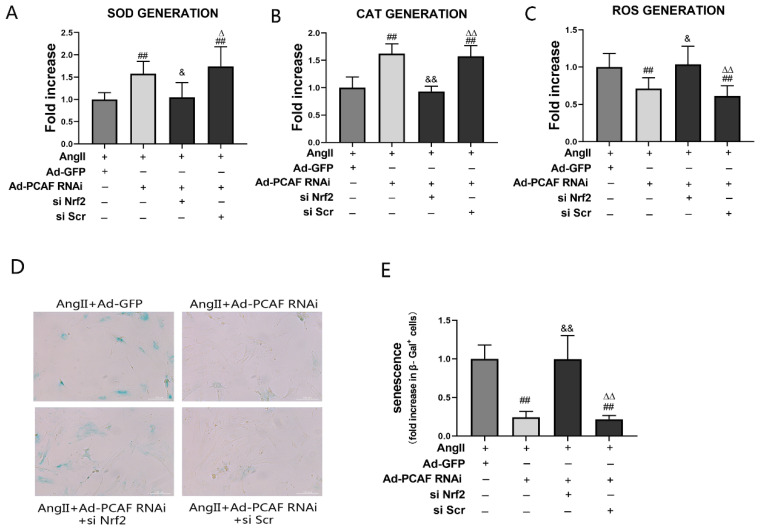
Nrf2 silencing aborted the anti-senescence effect of downregulation of PCAF in ECs. (**A**) Commercial kits to detect SOD activity in ECs (*n* = 5). (**B**) Commercial kits to detect CAT activity in ECs (*n* = 5). (**C**) Commercial kits to detect ROS activity in ECs (*n* = 5). (**D**,**E**) β-Galactosidase activity assay was used to reflect the senescence of ECs. Senescent ECs are marked in blue; the scale bar is 200 μm, 200× (*n* = 5). ## *p* < 0.01 vs. AngII + Ad-GFP group; & *p <* 0.05, && *p <* 0.01 vs. AngII + Ad-PCAF RNAi group; Δ *p* < 0.01, ΔΔ *p* < 0.01 vs. AngII + Ad-PCAF RNAi + si Nrf2 group.

To further verify that the activation of Nrf2 and the expression of its target genes play a role in downregulating PCAF to resist senescence of ECs, we constructed Nrf2 small interfering RNA (siRNA) for experiments. As shown in Supplementary Figure S1, the expression of Nrf2 and its downstream HO-1 and NQO1 in ECs decreased significantly after Nrf2 silencing. Figure 6A–C show that Nrf2 siRNA transfection could significantly decrease the levels of SOD and CAT, and increase the level of ROS, compared with those in the AngII + Ad-PCAF RNAi group (all *p* < 0.05). It was suggested that silencing of Nrf2 remarkably weakened the antioxidative stress effect of downregulation of PCAF. Meanwhile, the senescence of ECs in the AngII + Ad-PCAF RNAi + Nrf2 siRNA transfection group was significantly increased compared with that in the AngII + Ad-PCAF RNAi group (*p* < 0.05) (Figure 6D,E). 

## 3. Discussion

In recent years, cardiovascular and cerebrovascular diseases have become the leading causes of death worldwide [32]. Vascular aging is considered as one of the main risk factors for adverse cardiovascular and cerebrovascular events [33]. Vascular aging is a multifaceted and complex process, which is mainly manifested by changes in the structure and function of the vascular system associated with senescence of vascular cells. In this study, we provide the first evidence for PCAF mediating vascular aging. Our results showed that the expression of PCAF was associated with age and aging, and remarkably increased expression of PCAF was present in human atherosclerotic coronary artery. Downregulation of PCAF could reduce angiotensin II (AngII)-induced senescence of rat aortic endothelial cells (ECs) in vitro. In addition, inhibition of PCAF with garcinol alleviated AngII-induced vascular senescence phenotype in mice. Furthermore, we also demonstrated that PCAF interacted with Nrf2 and the anti-senescence activity of downregulation of PCAF was mediated by the Nrf2 signaling pathway.

Age has been used as an indicator of physical aging for many years. It is generally believed that in the human vasculature, senescence will gradually become obvious with age [11]. As Sir William Osler in the late 19th century stated, “you are only as old as your arteries”. Based on the above characteristics, we detected the expression of PCAF in the coronary arteries of 44 heart transplant patients and found that PCAF was positively correlated with the patient’s age (Figure 1B). It is worth noting that in patients with premature aging, age does not match the degree of vascular aging. Therefore, in the analysis of the correlation between age and the expression level of PCAF in coronary vessels, we first excluded specimens from patients with premature aging. Besides age, atherosclerosis is closely related to vascular senescence [34]. Cardiovascular risk factors accelerate the normal biological senescence of vascular, leading to telomere attrition of vascular cells, accumulation of DNA damage and increased oxidative stress [35,36]. These pathological processes cause damage to normal vascular cell function and aggravated inflammation in aging and in atherosclerotic plaques. Cellular dysfunction leads to changes in extracellular matrix proteins, contributing to vascular stiffness and loss of elasticity, and increased inflammation promotes atherosclerosis. In addition, atherosclerosis may also directly lead to accelerated vascular senescence, because inflammation and ROS promote stress-induced premature aging, and cell senescence was also observed in patients with atherosclerosis [27]. Therefore, atherosclerosis is a disease of both organismal aging and cellular aging. In this study, we found that the expression of PCAF was significantly increased in the coronary artery with atherosclerosis, and the levels of aging-related proteins such as p53, p21, p16, ICAM-1 and VCAM-1 were also markedly enhanced. In addition, we also found that the antioxidant activity driven by the Nrf2 pathway was significantly abated in the atherosclerosis coronary arteries. These results further proved the link between human vascular aging and the expression of PCAF.

Proper Ang II concentration is necessary to maintain the normal physiological functions of the cardiovascular system, but excessive Ang II accumulation in the body will promote the occurrence and development of a variety of cardiovascular diseases [37]. In recent years, AngII as an effective inducer of vascular senescence in vivo and in vitro has been reported in many studies [38,39]. After senescence induced by AngII, vascular cells not only reveal cell proliferation and differentiation disorders, but also manifest a series of specific aging-related phenotypic traits [40]. Enhanced SA-β-gal activity is the most significant feature of senescent vascular systems and cells [41]. In addition, senescent cells also have an active senescence-related secretory phenotype (SASP) and increased expression of cell-cycle-related proteins (including p53, p21 and p16) [42,43,44]. The main components of SASP are inflammation factors, chemokines, adhesion molecules and proteases. Besides, different type of cells also have certain differences in SASP composition after aging. Our results in vitro showed that the expression of PCAF was significantly increased in AngII-induced senescent ECs. Meanwhile, the β-galactosidase activity, the expression of inflammatory factors IL-6 and TNF-α, and the abundance of adhesion molecules VCAM-1 and ICAM-1 all increased significantly. However, downregulation of PCAF could reverse the aging phenotype. Similar findings were noticed in vivo experiments.

Nrf2 is mainly retained in the cytoplasm by binding to the negative regulator keap1. However, under the stimulation of ROS or other senescence-inducing factors, Nrf2 could dissociate from keap1 and be transferred to the nucleus where it binds to antioxidant response elements (ARE), and then promote the transcription and expression of antioxidant factors, and ultimately reduce oxidative stress damage and inhibit senescence [45,46]. In addition, the activation of Nrf2 also prevents the accumulation of ROS in cells by regulating the expression of NQO1. An important finding in our research is that downregulation of PCAF was directly related to the activation of Nrf2-driven antioxidant defense pathways. In this study, we found that in senescent ECs, mice vascular tissues and atherosclerotic human coronary arteries, the upregulation of PCAF was associated with the decrease in the abundance of nucleus Nrf2. After treatment with Ad-PCAF RNAi or PCAF inhibitors, the accumulation of nuclear Nrf2 was enhanced and the antioxidant enzymes were subsequently increased. At the same time, pre-transfection with Nrf2 siRNA reverse the effect of Ad-PCAF RNAi in ECs. In addition, the results of co-immunoprecipitation showed that there was a direct interaction between PCAF and Nrf2 in ECs. These results indicate that PCAF was involved in the regulation of Nrf2 pathway, and the anti-vascular senescence effect of PCAF downregulation was mainly exerted by the enhancement of antioxidant activity driven by the activation of the Nrf2 pathway.

In conclusion, the expression of PCAF was significantly enhanced in aging human coronary arteries, Ang II induced senescent mice aorta and rat ECs. Downregulation of PCAF exerts an anti-aging effect by promoting the activation of Nrf2 to suppress oxidative stress damage. Thus, PCAF could be an attractive potential target for the prevention and treatment of vascular aging-related diseases.

## 4. Materials and Methods

### 4.1. Materials and Agents

Angiotensin II (Ang II) was purchased from Sigma-Aldrich (#05-23-0101, Saint Louis, MO, USA). Cell Counting Kit 8 (CCK-8) was obtained from Dojindo Molecular Technologies (#CK04, UMAMOTO KEN, Japan). Senescence β-Galactosidase (SA-β-Gal) Staining Kit was bought from Beyotime Biotechnology (#C0602, Shanghai, China). Reactive oxygen species (ROS) assay kit, Superoxide Dismutase (SOD) assay kit and Catalase (CAT) assay kit were obtained from Nanjing Jiancheng Bioengineering Institute (#E004-1-1, #A001-3, # A007-1-1, Nanjing, China). The primary antibodies against glyceraldehyde-phosphate dehydrogenase (GAPDH) were obtained from Servicebio (#GB11002, #GB12002, Wuhan, China). Nuclear factor erythroid-2-related factor 2 (Nrf2) were obtained from Proteintech Group (#66504-1, Wuhan, China). Antibodies against PCAF (#3378), tumor suppressor protein p53 (p53) (#2524) and Heme Oxygenase-1 (HO-1) (#43966) were obtained from Cell Signaling Technology (Danvers, MA, USA). Antibodies against PCAF (#sc-13124), NAD(P)H quinone oxidoreductase-1 (#sc-376023), Cyclin-dependent kinase inhibitor 1A (p21) (#sc-6246), cyclin-dependent kinase inhibitor 2A (p16) (#sc-1661), intercellular cell adhesion molecule-1 (ICAM-1) (#sc-8439) and vascular cell adhesion molecule 1 (VCAM-1) (#sc-13160) were obtained from Santa Cruz Biotechnology (Santa Cruz, CA, USA). Rat Nrf2 small interfering RNA (siRNA) and control siRNA were purchased from GenePharma (Huzhou, China).

### 4.2. Construction of Adenoviral Vectors and Nrf2 siRNA

The specific method for adenovirus construction in this study is the same as described previously [47]. The siRNA sequence of the rat PCAF gene was designed and synthesized by Genechem (Shanghai, China). A scrambled siRNA was used as a negative control. The sense siRNA sequence of PCAF is 5′-GACAAACTGCCTCTTGAGAAA-3′, and the sense siRNA sequence for the control is 5′-TTCTCCGAACGTGTCACGT-3′. Adenovirus-encoded siRNA against PCAF (Ad-PCAF-RNAi) and control (Ad-GFP) were produced by co-transfecting HEK293 cells according to standard protocols. Nrf2 siRNA was designed and synthesized by Hippo Biotechnology (Huzhou, China). The core sequence of rat Nrf2 gene is 5′-GCAGCAUACAGCAGGACAUTT-3′, and 5′-UUCUCCGAACGUGUCACGU-3′ for the control.

### 4.3. Primary Vascular Endothelial Cells Culture and Adenovirus Transfection

Rat aortic ECs were obtained from iCell Bioscience (#RAT-iCell-c003, Shanghai, China). The third to fifth passage of ECs was used for the following experiments. ECs were cultured with endothelial cell growth medium containing 10% fetal bovine serum (FBS). For adenovirus transfection, cells with a density of about 50% confluence were incubated with Ad-PCAF-RNAi or Ad-GFP at a multiplicity of infection (MOI) of 50 for 4 h. Then, the subsequent experiments were performed.

The ECs were randomly divided into four groups: control group, AngII group (continuously induced with 2 μM AngII for 24 h), AngII + Ad-GFP group (transfected with Ad-GFP adenovirus and then induced with 2 μM AngII for 24 h), AngII + Ad-PCAF RNAi group (Ad-PCAF RNAi adenovirus was transfected and then induced with 2 μM AngII for 24 h).

### 4.4. SA-β-Gal Staining

After processed as required, the cells were stained using a senescence-β-galactosidase (SA-β-gal) staining kit. The specific steps were implemented according to the kit instructions. After staining, five representative pictures were randomly taken from each group, and the ratio of the number of positive cells to total cells was calculated. 

### 4.5. Western Blot

Total cellular proteins were extracted using a RIPA lysis buffer (Beyotime Biotechnology, #P0013B, Shanghai, China). Cell cytosolic and nuclear lysate was extracted with nuclear and cytoplasmic protein extraction kit (Beyotime Biotechnology, #P0027, Shanghai, China). After quantifying with BCA kit, an equivalent amount of 30 µg proteins were loaded into 10% SDS-polyacrylamide gel for electrophoresis. Then, the protein was transferred to polyethylene fluoride (PVDF) by transfer system. The membranes were thereafter blocked with 5% skim milk in tris-buffered solution containing 1% tween 20 (TBST) for 2 h. Next, the membrane was washed with PBST, and the corresponding primary antibodies were used to incubate the membrane overnight at 4 °C. After that, the membranes were incubated with the secondary antibodies at room temperature for 1 h. Finally, the membranes were incubated with ECL solution, and visualization of the blots was achieved with Bio-Rad ChemiDoc Touch Imaging System.

### 4.6. Immunofluorescence Staining

The ECs were seeded in 6-well plates containing coverslips. Following different treatments, the cells were fixed with 4% paraformaldehyde for 15 min, and then incubated with 0.3% Triton for 10 min. Next, the slides were blocked with 5% goat serum for 1 h at room temperature, and then incubated overnight with primary antibodies at 4 °C. After that, the slides were incubated with fluorescently labeled secondary antibody for 1 h at room temperature. The nucleuses were stained with DAPI, and finally, anti-fluorescent quencher was added and coverslips were mounted. Images were captured using a fluorescence microscope. Experiments were repeated three times.

### 4.7. Detection of SOD, CAT and ROS

The cells were collected following pretreatment, and the commercial kits (Nanjing Jiancheng Bioengineering Institute, Nanjing, China) for SOD (#A001-3), CAT (# A007-1-1) and ROS (#E004-1-1) were used to analyze the activity levels. All procedures were performed in accordance with the manufacturer’s instructions. Three independently repeated experiments were performed.

### 4.8. Co-Immunoprecipitation

The co-immunoprecipitation (IP) assay was performed as described [48]. Briefly, the cells were harvested and treated with an appropriate amount of cell IP lysis buffer containing protease inhibitor. Then, the cells were lysed on ice for 30 min and centrifuged at 12,000× *g* for 30 min. The supernatants were collected. Next, a small amount of lysate was taken for Western blot analysis, and the remaining cell lysate was incubated with the appropriate amount of antibody and corresponding protein A/G-beads under slow shaking at 4 °C overnight. After the immunoprecipitation reaction, the cell lysate was centrifuged at 3000× *g* at 4 °C for 5 min. The supernatant was removed and the beads were washed with 1 mL lysis buffer 3–4 times. Next, 2 × SDS loading buffer was added and the lysate was boiled for 10 min. Finally, the Western blotting analysis was performed as abovementioned.

### 4.9. Animal Studies

The protocol for animal care was conformed to the Guide for the Care and Use of Laboratory Animals (the National Academies Press, 2011) and the experiment was approved by the Animal Care and Use Committee of Renmin Hospital of Wuhan University (approval no.: WDRM20210104). For vascular aging studies, C57BL/6 mice (male, 20–25 g, 1.5–2 months) were randomly divided into three groups: control group, Ang II infusion group (AngII group) and AngII + Garcinol (Sigma-Aldrich Co., Saint Louis, MO, USA) intraperitoneal injection group (Garcinol group). In short, mice were anesthetized with sodium pentobarbital; then, an Alzet osmotic micropump containing only saline or Ang II (1.5 mg/kg/d) dissolved in saline was implanted subcutaneously. Mice were intraperitoneally injected with garcinol (0.5 mg/kg/d) or equivalent vehicle after osmotic minipump implantation for 28 consecutive days. Based on previous data, the dose of garcinol in this study was expected to be non-toxic [29,49].

### 4.10. Human Vascular Specimen Collection and Immunohistochemical Staining

The coronary vascular specimens of humans involved in this study were all from patients undergoing heart transplantation in the Cardiac Surgery Department of Renmin Hospital of Wuhan University. The project was approved by the Ethics Committee of Renmin Hospital of Wuhan University (approval no.: WDRY2019-K231), and all the participants signed the informed consent form. The proximal of the right coronary artery specimens of 44 patients were made into tissue chips for immunohistochemical staining. The clinical information of the patients enrolled in this study are summarized in Supplementary Table S1. At the same time, the relevant clinical data were collected and analyzed. The tissue chips were sectioned at 4 μm and placed on the glass slide to dry. The antibody against PCAF was used for immunohistochemical staining according to the protocol. The average optical density in each specimen was analyzed using Image J software v1.51 (National Institutes of Health, Bethesda, MD, USA). Then, Pearson’s correlation was used to analyze the relationship between PCAF expression and patient age.

### 4.11. Statistical Analysis

Experimental results were presented as mean ± standard deviation (SD) and the statistical analysis was performed with Statistical Product and Service Solutions 22.0 software. Student’s *t*-test was used for comparisons between two groups, analysis of variance (ANOVA) was used for multiple comparisons, and Tukey’s test was used for post-hoc tests. A *p* value less than 0.05 was considered statistically significant.

## Data Availability

All data utilized in this study are included in this article, and all data supporting the findings of this study are available on reasonable request from the corresponding author (C.X.).

## Supplementary Materials

The following supporting information can be downloaded at: https://www.mdpi.com/article/10.3390/ijms232012574/s1.
