# Supplementary material for: Downregulation of P300/CBP-Associated Factor Protects from Vascular Aging via Nrf2 Signal Pathway Activation
Source: Int J Mol Sci. 2022 Oct 20;23(20):12574. doi: 10.3390/ijms232012574 (PMC9603891; doi:10.3390/ijms232012574)
Supplement: Supplementary file 1 [file ijms-23-12574-s001.zip › ijms-1929803-supplementary.pdf]

Table S1. The clinical information of the patients enrolled in this study

	Name	Gender	Age (years)	Main Clinical Diagnosis	Operative Methods	PCAF Expression (optical density)
1	*LM*	female	15	Complex cyanotic congenital heart disease	heart transplantation	0.264
2	*FJQ*	male	18	dilated cardiomyopathy	heart transplantation	0.268
3	*ZX*	male	30	dilated cardiomyopathy	heart transplantation	0.468
4	*ZYQ*	male	33	dilated cardiomyopathy	heart transplantation	0.273
5	*ZCQ*	male	34	dilated cardiomyopathy	heart transplantation	0.38
6	*ZJ*	male	35	dilated cardiomyopathy	heart transplantation	0.191
7	*CZH*	male	37	dilated cardiomyopathy	heart transplantation	0.231
8	*LYS*	male	38	dilated cardiomyopathy	heart transplantation	0.21
9	*ZWL*	male	40	dilated cardiomyopathy	heart transplantation	0.529

10	*ZC*	male	42	heart failure	heart-lung transplantation	0.426
11	*LSB*	male	42	dilated cardiomyopathy	heart transplantation	0.429
12	*WBJ*	male	42	ischemic cardiomyopathy	heart transplantation	0.354
13	*GMZ*	male	43	dilated cardiomyopathy	heart transplantation	0.506
14	*HXR*	female	43	ischemic cardiomyopathy	heart transplantation	0.561
15	*HFX*	male	44	ischemic cardiomyopathy	heart transplantation	0.736
16	*CJY*	male	45	ischemic cardiomyopathy	heart transplantation	0.587
17	*ZCH*	male	45	dilated cardiomyopathy	heart transplantation	0.697
18	*WLQ*	male	48	ischemic cardiomyopathy	heart transplantation	0.763
19	*HW*	male	48	ischemic cardiomyopathy	heart transplantation	0.769
20	*CHR*	male	49	Eisenmenger syndrome	heart transplantation	0.728
21	*LSX*	male	49	dilated cardiomyopathy	heart transplantation	0.703
22	*GXF*	male	49	dilated cardiomyopathy	heart transplantation	0.934

23	*WWY*	female	50	ischemic cardiomyopathy	heart transplantation	0.801
24	*RAM*	female	50	dilated cardiomyopathy	heart transplantation	0.794
25	*JWD*	male	51	ischemic cardiomyopathy	heart transplantation	0.905
26	*FDL*	male	52	dilated cardiomyopathy	heart transplantation	0.869
27	*CJY*	female	52	valvular heart disease	heart transplantation	0.825
28	*SSL*	female	52	dilated cardiomyopathy	heart transplantation	0.874
29	*LJX*	female	53	heart failure	heart transplantation	0.786
30	*ZDZ*	female	53	ischemic cardiomyopathy	heart transplantation	0.791
31	*XHK*	male	54	ischemic cardiomyopathy	heart transplantation	0.859
32	*MCX*	male	55	dilated cardiomyopathy	heart transplantation	0.874
33	*LGY*	male	56	dilated cardiomyopathy	heart transplantation	0.783
34	*WZY*	male	56	dilated cardiomyopathy	heart transplantation	0.907
35	*ZLF*	male	57	dilated cardiomyopathy	heart transplantation	0.74

36	*YWZ*	male	58	dilated cardiomyopathy	heart transplantation	0.869
37	*LJS*	male	59	dilated cardiomyopathy	heart transplantation	0.937
38	*CE*	male	59	dilated cardiomyopathy	heart transplantation	0.823
39	*YAP*	female	59	ischemic cardiomyopathy	heart transplantation	0.841
40	*ZYM*	male	59	ischemic cardiomyopathy	heart transplantation	0.874
41	*GSQ*	male	60	heart failure	heart transplantation	0.976
42	*SCR*	female	60	dilated cardiomyopathy	heart transplantation	0.713
43	*WKF*	female	60	dilated cardiomyopathy	heart transplantation	0.883
44	*ZQG*	male	61	dilated cardiomyopathy	heart transplantation	0.893

Supplementary Figure S1

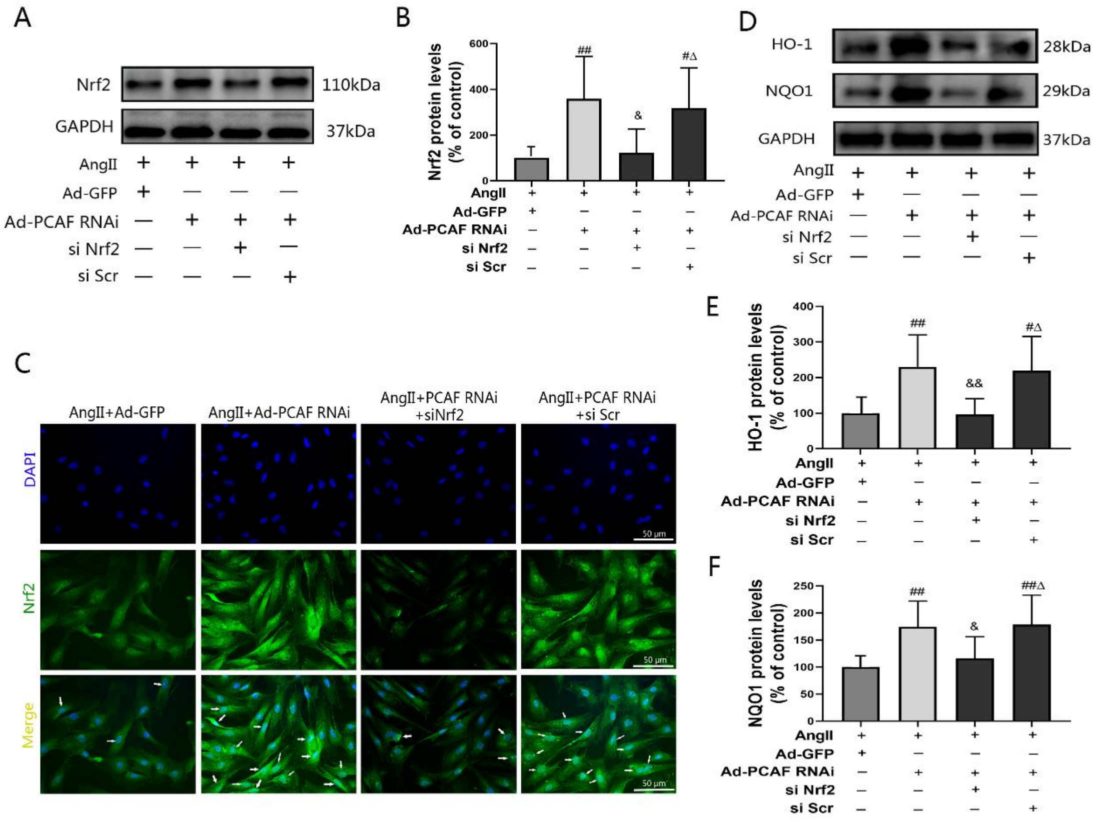

Supplementary Figure S1 The expression of Nrf2 and its downstream HO-1 and NQO1 in ECs decreased significantly after Nrf2 silencing. (A-B) Nrf2 small interfering RNA (siRNA) inhibits the expression of Nrf2 in ECs, detected by western blot (n=6). (C) Immunofluorescence detection of Nrf2 distribution in each group. The scale bar is 50 μm, 400× (n=3). (D-F) Western blot to determine the expression of HO-1 and

NQO1 in each group (n=6). $^{\#}p < 0.05$, $^{\#\#}p < 0.01$ vs. AngII+Ad-GFP group; $^{\&}p < 0.05$, $^{\&\&}p < 0.01$ vs. the AngII induction with Ad-PCAF RNAi group (AngII+Ad-PCAF RNAi group); $^{\triangle}p < 0.05$ vs. the AngII induction with Ad-PCAF RNAi group and Nrf2 small interfering RNA (AngII+Ad-PCAF RNAi+si Nrf2 group).
